# Bilateral Systemic Artery-to-Pulmonary Vessel Fistulas Following Video-Assisted Thoracoscopic Surgery for Primary Spontaneous Pneumothorax: A Case Report

**DOI:** 10.7759/cureus.87862

**Published:** 2025-07-13

**Authors:** Daiki Mihara, Hiroyuki Tao

**Affiliations:** 1 Department of Thoracic Surgery, Japanese Red Cross Society Himeji Hospital, Himeji, JPN

**Keywords:** pneumothorax, sapvf, systemic artery-to-pulmonary vessel fistula, vats, video-assisted thoracic surgery

## Abstract

Systemic artery-to-pulmonary vessel fistula (SAPVF) is a rare vascular anomaly, with acquired forms typically arising secondary to intrathoracic inflammation, infection, trauma, or thoracic surgery. We report a case of a 21-year-old man with a history of bilateral video-assisted thoracoscopic surgery (VATS) bullectomy for primary spontaneous pneumothorax who developed bilateral SAPVF. Contrast-enhanced computed tomography revealed abnormally dilated pulmonary vessels with systemic arterial communications in both lungs, corresponding to previous surgical incision sites. On the left side, SAPVF developed despite the surgical wound separated from the lung by oxidized regenerated cellulose sheets. Due to multiple systemic feeding arteries, embolization was considered ineffective, and surgery was avoided owing to the high risk of postoperative reformation. The patient has been managed conservatively, with no progression observed over a two-year follow-up period. This case highlights that SAPVF can occur even after minimally invasive surgery such as VATS, emphasizing the need for careful follow-up and further evaluation of optimal preventive strategies.

## Introduction

A systemic artery-to-pulmonary vessel fistula (SAPVF) is a rare vascular malformation characterized by abnormal communication between systemic arteries and pulmonary arteries or veins. SAPVF can arise through pleural adhesions resulting from inflammatory or neoplastic processes, allowing systemic arteries to invade the pulmonary parenchyma without traversing the capillary bed [1]. SAPVF is classified as congenital or acquired. The acquired SAPVF arises secondary to intrathoracic inflammation, infection, trauma, or thoracic surgery. Among postoperative cases, most were associated with prior coronary artery bypass grafting (CABG) or lung resection via thoracotomy [1,2]. In recent years, a few SAPVF cases have been reported after video-assisted thoracoscopic surgery (VATS), a minimally invasive surgical approach, but such reports remain exceedingly rare [3,4]. Herein, we report an infrequent case of bilateral SAPVF following bilateral VATS for primary spontaneous pneumothorax. To our knowledge, this is the first case of bilateral SAPVF occurring after VATS.

## Case presentation

**Figure 1:**
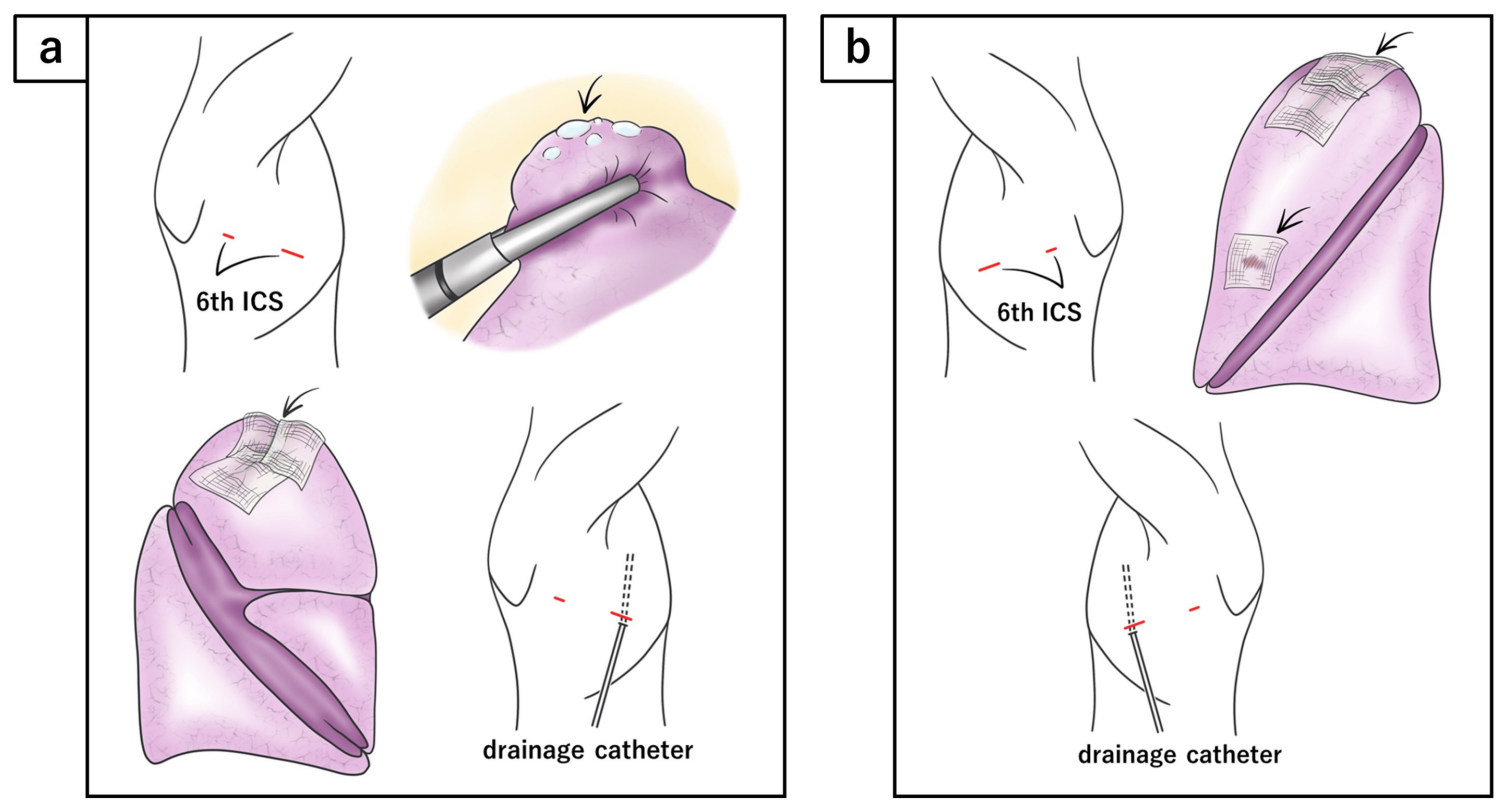
Details of previous operations (a) Right-sided operation at age 17: A 2 cm anterior axillary incision and a 5 mm posterior axillary incision were made at the sixth intercostal space. A wound protector was used for the anterior incision, and a 5 mm port was used for the posterior incision. Apical bullae were resected using three stapler firings. The staple line was covered with three sheets of oxidized regenerated cellulose (SURGICEL®). No polyglycolic acid sheet was used, and pleural abrasion was not performed. A 6.5 mm multi-channel drainage catheter was placed. The muscular layer was closed using 2-0 Polysorb (Covidien), and the subcutaneous layer was sutured with 4-0 polydioxanone (PDS II; Ethicon). The parietal pleura was not sutured. (b) Left-sided operation at age 19: The surgical approach and technique were similar to the right side. Bullae were resected, and the staple line was covered with oxidized regenerated cellulose. In addition, a sheet was applied to the site of lung injury located directly beneath the incision. The muscular layer was closed using 3-0 PDS, and the subcutaneous layer was sutured with 4-0 PDS. The parietal pleura was not sutured. All illustrations were created by the authors using Procreate (Savage Interactive, Hobart, Tasmania, Australia) on an iPad.

A 21-year-old man was found to have an abnormality on a chest radiograph during a medical checkup. He was asymptomatic, and laboratory findings were within normal limits. He was a never-smoker. He had a history of bilateral VATS bullectomy performed at ages 17 and 19 for primary spontaneous pneumothorax. Both procedures involved apical bullectomy and coverage of the staple line with oxidized regenerated cellulose. For the initial right-sided pneumothorax, a tube thoracostomy was performed two days prior to surgery. In contrast, the subsequent left-sided pneumothorax was treated surgically without preceding drainage. Pleural abrasion was not performed in either surgery. In both surgeries, the chest tube was removed on postoperative day 1, and the drain site was closed with compression only. The postoperative course was uneventful in each case. Surgical details are illustrated in the figures below (Figure 1a-1b).

**Figure 2:**
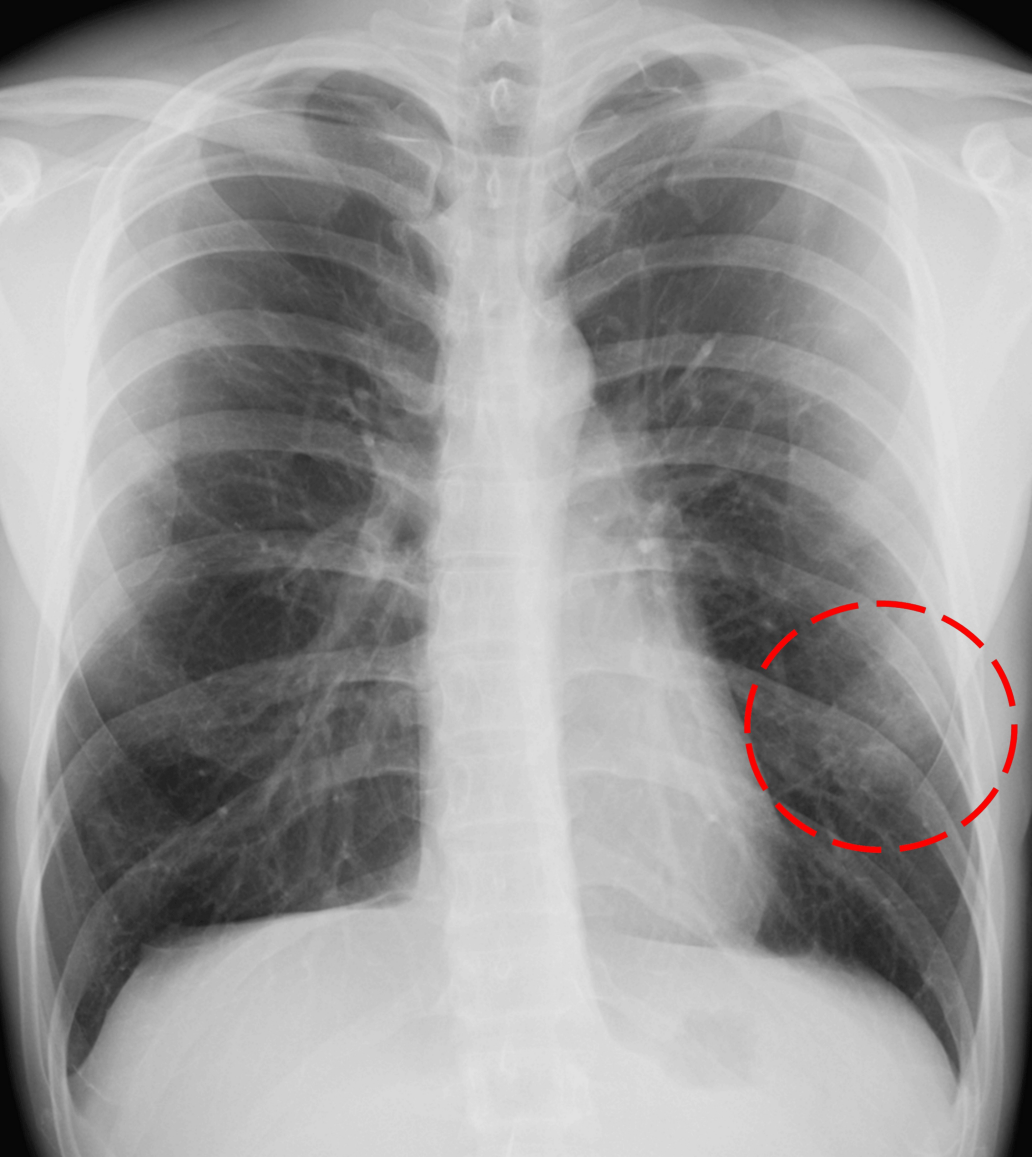
Chest radiograph The chest X-ray showed an ill‑defined focal opacity in the lower left lung field.

**Figure 3:**
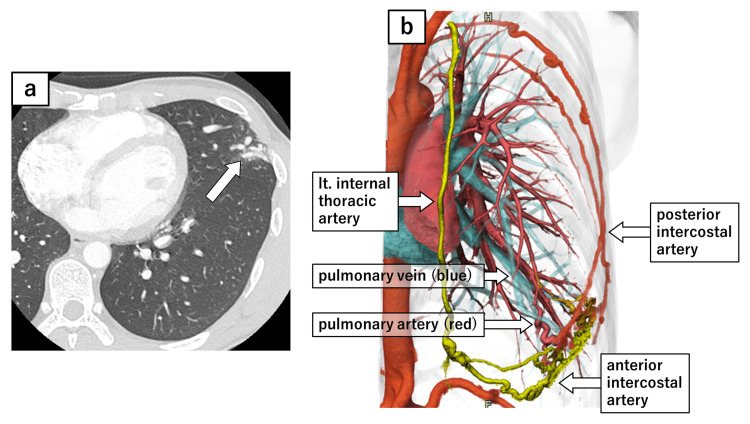
Contrast enhanced CT of the left side and 3D reconstruction. (a) Contrast-enhanced CT revealed significantly dilated pulmonary vessels in the posterior lingular segment (S5), which were connected to arteries in the chest wall. (b) 3D-CT reconstruction, created using SYNAPSE VINCENT® (Fujifilm Medical Co., Ltd., Japan), revealed that both the pulmonary artery and vein were connected to systemic arteries at the surgical wound, including the posterior intercostal arteries and the anterior intercostal artery, which is a branch of the internal thoracic artery. CT: computed tomography, 3D: three dimensional

**Figure 4:**
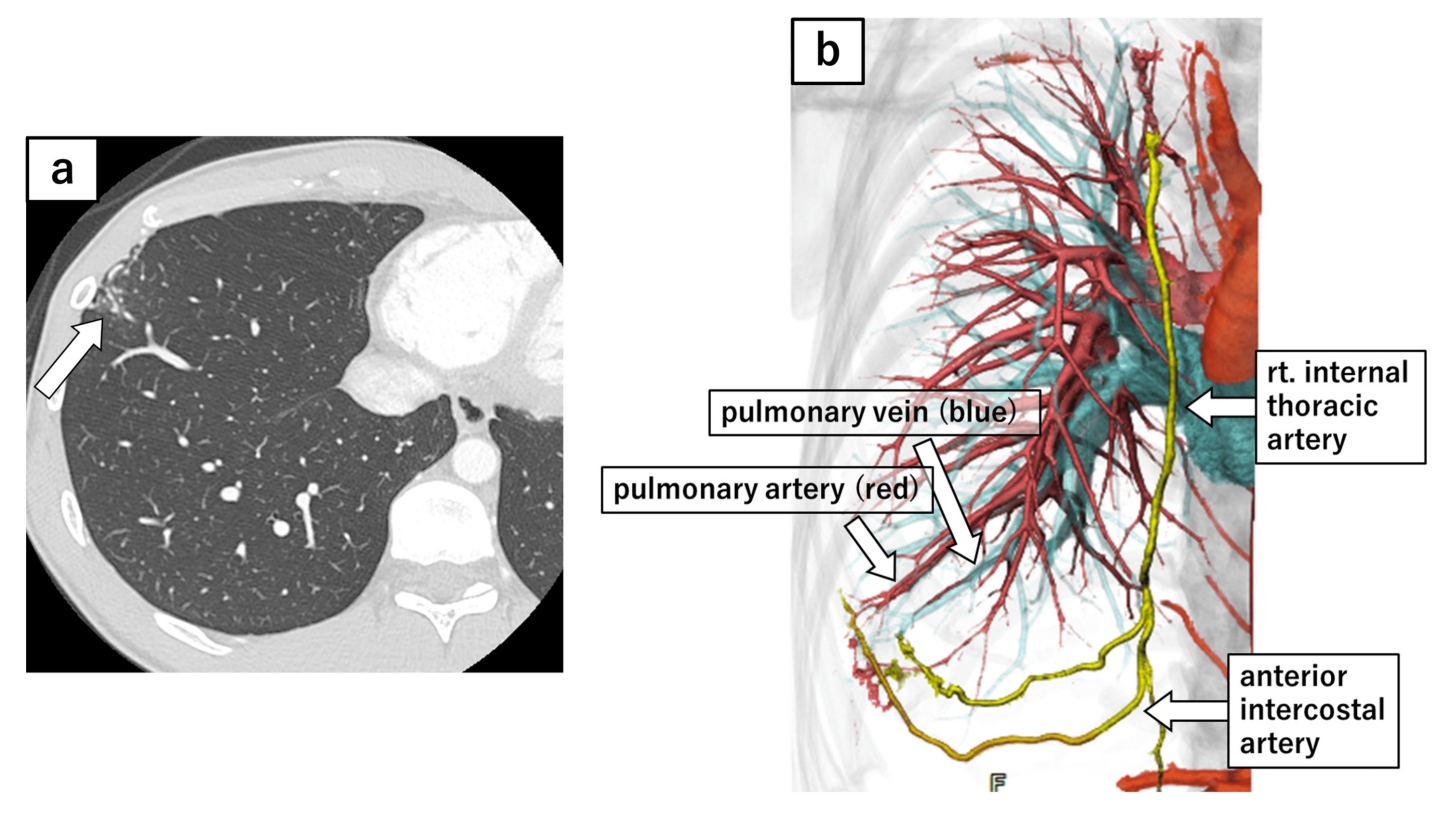
Contrast enhanced CT of the right side and 3D reconstruction (a) Contrast-enhanced CT demonstrated dilated pulmonary vessels in the right anterior basal segment (S8), which were connected to chest wall arteries, including both the anterior and posterior intercostal arteries. (b) 3D-CT reconstruction, created using SYNAPSE VINCENT® (Fujifilm Medical Co., Ltd., Japan), revealed that both the pulmonary artery and vein were connected to systemic arteries at the surgical wound, including the anterior intercostal arteries, which are branches of the internal thoracic artery. Although the posterior intercostal artery was identified on contrast-enhanced CT, it was not visualized on 3D-CT reconstruction. CT: computed tomography, 3D: three dimensional

The chest X-ray showed an ill-defined focal opacity in the lower left lung field (Figure 2), and contrast-enhanced chest computed tomography (CT) revealed dilated pulmonary vessels in the peripheral lingular segment, with fistulous communications involving the intercostal arteries (Figure 3a-3b). These findings were consistent with the previous surgical incision site. Similar vascular findings were observed in the lower lobe of the right lung, corresponding to the previous incision site (Figure 4a-4b). Subsequently, he was diagnosed with bilateral SAPVF.

Management and follow-up

Because of the presence of multiple feeding arteries on both sides, endovascular embolization was considered ineffective. Furthermore, the SAPVF on the left side of this patient developed despite the surgical wound supposedly being separated from the lung by oxidized regenerated cellulose; concerns regarding the reformation of SAPVF after surgical resection were also considered. Since the patient was asymptomatic, we opted for careful observation with regular chest CT. No obvious progression was observed during the two-year follow-up period.

## Discussion

SAPVF is a rare vascular malformation that causes abnormal communication between the systemic arteries and the pulmonary circulation. Acquired SAPVF typically arises due to the development of neovascularization following adhesions between the lung and chest wall, secondary to inflammation, trauma, or surgery [1,2]. Most previously reported cases of postoperative SAPVF have occurred following CABG or lung resection via thoracotomy [1,2], and there are extremely limited reports of cases occurring following VATS, which is considered to be less invasive and results in milder adhesions compared to conventional open thoracotomy [3,4]. However, there have also been reports of SAPVF occurring after thoracic drainage with a smaller incision [1], indicating that SAPVF can occur regardless of the type of thoracic wall injury. Previous reports of iatrogenic or post-traumatic SAPVF have shown a median interval of 4.5 years (range, 1-25 years) between the inciting event and diagnosis [1,3-7]. After CABG, this interval ranges from 2 months to 16 years [2]. No large-scale studies have evaluated the incidence of postoperative SAPVF, and its true prevalence remains unknown. However, follow-up after surgery for spontaneous pneumothorax is generally short, raising the possibility of undetected cases. Considering the reported median interval, regular imaging follow-up at least five years postoperatively may help facilitate earlier detection. As dilated pulmonary vessels can sometimes be identified on non-contrast CT or even chest radiographs, contrast-enhanced CT may not be indispensable for detection. Moreover, symptomatic SAPVF is frequently diagnosed many years after the inciting event, with a median interval of 17 years (range, 2-25 years) [1,4,5-7], suggesting that early identification based on imaging findings could potentially prevent progression to symptomatic or more severe disease.

On the other hand, in our case, the right-sided VATS was performed two years earlier than the left, yet the SAPVF on the right appeared milder. A similar pattern was observed in a report by Shimmyo et al. [3], where SAPVF occurred only on the left side despite bilateral surgery. These findings suggest that factors other than the time interval may contribute to the development of SAPVF. In our case, there were no substantial differences in surgical technique or perioperative management between the two procedures. However, inadvertent injury to the visceral pleura of the lingular segment during the left-sided operation may have promoted stronger adhesions, contributing to the more prominent SAPVF. Interestingly, SAPVF was also localized to the lingular segment in reports by Shimmyo et al. [3] and Kuramochi et al. [4], suggesting that this region may be more prone to adhesion and fistula formation due to anatomical factors or the typical incision site in apical bullectomy.

Pleural abrasion is recommended by the American College of Chest Physicians and British Thoracic Society guidelines for the management of spontaneous pneumothorax [8,9]. However, in Japan, bullectomy combined with covering of the visceral pleura has become the standard treatment for spontaneous pneumothorax. In contrast, pleural abrasion or pleurectomy is performed in only about 1% of cases [10,11]. The covering technique is performed to thicken the visceral pleura and/or prevent adhesion to the chest wall. At our institution, bullectomy combined with covering of the visceral pleura is the standard procedure, and no intervention is performed on the parietal pleura. In the present case, oxidized regenerated cellulose (SURGICEL®) was used, but SAPVF still developed. Although it remains unclear whether the oxidized cellulose sheets were definitively interposed between the lung and the surgical wound, this case suggests that the coverage method used may have been insufficient to prevent adhesions. Oxidized regenerated cellulose is known to be effective in preventing intrathoracic adhesions after lung resection [12]. Compared to polyglycolic acid sheets, it is considered to promote pleural thickening and suppress adhesion formation more effectively [13]. However, its effect may be limited by incomplete coverage, and excessive use may interfere with lung re-expansion. As shown in this case, it may not always be possible to completely prevent adhesions. In the future, optimization of the coverage area and application method is expected to improve outcomes. In addition, suturing the parietal pleura without defects may help prevent SAPVF by preventing adhesions between the lung and chest wall and preventing direct contact between chest wall arteries and pulmonary vessels [4].

Regarding treatment strategies, conservative observation may be considered in asymptomatic cases [14]. However, complications such as hemoptysis [1,4,15,16], heart failure or pulmonary hypertension [5,17], and chronic pain [6] have been reported as manifestations of SAPVF. Furthermore, extensive pleural adhesions and abnormal neovascularization associated with SAPVF may complicate future thoracic interventions by increasing the risk of intraoperative bleeding or technical difficulties. So, many reported cases have undergone embolization or resection. In our case, multiple feeding arteries were involved, and given the low success rate of embolization and the high likelihood of re-adhesion or reformation of SAPVF following surgery, we decided to continue observation without invasive treatment unless symptoms develop or imaging shows progression.

## Conclusions

It is important to keep in mind that SAPVF can occur even after VATS, and regular imaging evaluations and careful follow-up are necessary after surgery. To more reliably prevent postoperative adhesions, it is desirable to select appropriate covering materials and consider the coverage area, as well as to accumulate knowledge on cases prone to SAPVF.
